# Evaluation of Data Acquisition Areas in Geotechnical Seismic Tests: Insights from Field Applications

**DOI:** 10.3390/s25061757

**Published:** 2025-03-12

**Authors:** Gunwoong Kim

**Affiliations:** Department of Geotechnical Engineering Research, Korea Institute of Civil Engineering and Building Technology, Goyang 10223, Gyeonggi, Republic of Korea; gwkim86@kict.re.kr

**Keywords:** geophysical testing, non-destructive testing, seismic testing, Vs profile, data acquisition, sub-surface information

## Abstract

Geotechnical field testing evaluates soil, rock, and groundwater conditions in their natural states, offering critical information about subsurface properties such as the density, strength, permeability, and groundwater flow. These tests are essential in ensuring the safety, reliability, and performance of civil engineering projects and are increasingly used for 3D geographical visualization and subsurface modeling. While point-based tests like the cone penetration test (CPT) and standard penetration test (SPT) are widely used, area-based methods such as the spectral analysis of surface waves (SASW) and electrical resistivity testing significantly enhance the accuracy of such models by providing broader coverage. Furthermore, these non-destructive techniques are particularly effective in identifying subsurface defects. This study focuses on analyzing the data acquisition areas of various field seismic tests, including SASW, downhole, crosshole, and suspension logging (PS logging). While other tests clearly define data acquisition areas based on their array paths, the SASW test posed challenges due to the complexity of data reconstruction. To address this, 69 datasets from four different sites were analyzed to predict the data acquisition areas for SASW as a function of depth. Moreover, a case study demonstrates the practical application of the SASW method in detecting cavities near a dam spillway. The findings of this research improve the understanding and interpretation of geotechnical seismic test data, enabling more precise geotechnical investigations and advancing the detection of subsurface defects using non-destructive methods.

## 1. Introduction

Geotechnical in situ testing involves determining and evaluating soil, rock, and groundwater conditions directly at the site in their natural states. Unlike laboratory tests that rely on random samples, in situ tests provide direct measurements and assessments of subsurface properties, such as the soil density, strength, permeability, and groundwater flow characteristics. These tests play a critical role in ensuring the safety, reliability, and performance of civil engineering projects.

**Figure 1:**
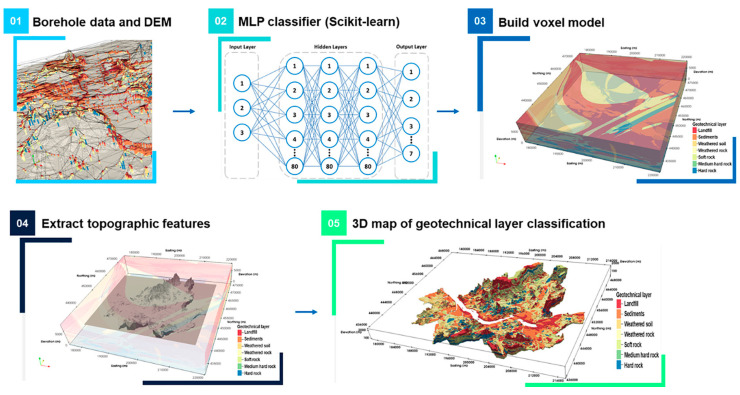
Conceptual architecture of proposed MLP-based 3D geotechnical layer classification model [1].

In addition to their importance in geotechnical design, geotechnical investigations are frequently utilized for 3D geographical visualization and subsurface modeling (Figure 1) [1]. Point-based test data, such as those obtained from the cone penetration test (CPT) and the standard penetration test (SPT), are commonly used to construct 3D models through kriging or interpolation methods. However, incorporating area-based data obtained from methods such as stratigraphic analysis, spectral analysis of surface waves (SASW), multi-channel analysis of surface waves (MASW), and electrical resistivity testing can significantly enhance the accuracy and reliability of these models [2]. Furthermore, these area-based tests are non-destructive testing methods, making them particularly useful in identifying defects or cavities effectively.

Among seismic testing methods, SASW and MASW have been widely employed for subsurface investigations due to their non-destructive nature and ability to cover large areas efficiently. A comparative study using the MSOR approach demonstrated that both methods were effective in subsurface characterization, with SASW providing higher-resolution depth profiling and MASW offering more intuitive and broader lateral coverage [3]. The selection between these methods depends on the project-specific requirements and site conditions.

Recent advancements in MASW have improved the accuracy through enhancements in inversion analysis, leading to the development of tools that utilize these improvements [4]. SASW has also been studied to ensure data reliability by investigating the reliability of phase angle differences [5]. Additionally, improvements in the inversion processes for SASW and MASW have been achieved through artificial intelligence applications. Studies have demonstrated that artificial neural networks and machine learning algorithms can enhance the accuracy of surface wave dispersion curve inversion, improving both the computational efficiency and the reliability of subsurface characterization [6,7].

For downhole and crosshole tests, research efforts have focused on refining the data interpretation techniques. The introduction of the spectral analysis of body waves (SABW) method has improved the accuracy of Vs profile comparisons by refining wave dispersion analysis [8]. This approach addresses the limitations associated with conventional techniques and enhances the reliability of downhole and crosshole seismic profiling.

While significant improvements have been made in data analysis techniques, ensuring that engineers can reliably utilize the analyzed data remains a challenge. Therefore, it is essential to clearly define their data acquisition areas. To efficiently perform 3D modeling and detect subsurface defects, it is crucial to understand the specific areas targeted by these tests. Tests such as the SPT and CPT, which rely on kinetic energy, have relatively straightforward data acquisition areas, as the tested area corresponds directly to the penetration depth. In contrast, geophysical testing techniques employing seismic waves require a deeper understanding of the wave path, as the data acquisition area extends beyond the penetration point.

Therefore, this study aims to define and analyze the data acquisition areas of various field seismic tests, including the SASW, downhole, crosshole, and suspension logging (PS logging) tests. The validity of these tests, including their measurement areas and applicability, has been thoroughly established through numerous studies over the years [9,10,11,12,13,14,15,16,17].

However, while downhole, crosshole, and suspension logging tests provide well-defined measurement areas based on their array paths, SASW poses challenges in accurately estimating its data acquisition area. Unlike another non-destructive surface wave test, MASW, which offers more intuitive lateral coverage, SASW requires a more complex data reconstruction process. Due to these challenges, this study focuses solely on the SASW method for surface wave testing to better understand its data acquisition characteristics. To address the uncertainty in data reconstruction for SASW, an analysis of the data measurement range was conducted using 69 test datasets obtained from four different sites.

Additionally, to compare the different tests, the four reviewed seismic tests were used to analyze the data acquisition areas as a function of the depth. Lastly, based on an understanding of the measurement area, a case study demonstrating the application of the SASW test in detecting cavities near a dam spillway is presented.

This research aims to improve the understanding and interpretation of geotechnical seismic test data. This study is expected to enhance practical applications, such as detecting defects using non-destructive methods and improving the precision of geotechnical investigations.

## 2. Geotechnical Seismic Testing Methodology

The geotechnical seismic tests commonly used in geotechnical investigations include SASW, downhole, crosshole, and PS logging. These geotechnical seismic tests measure the shear wave velocity, or Vs, value. The shear wave velocity is the most critical value for seismic design parameters. In addition, the shear wave velocity relates to the shear strength [18]. Furthermore, field measurements of the shear wave velocity are valuable in assessing soil resilience against liquefaction. For instance, studies have found correlations between the stress-normalized shear wave velocity and the cyclic resistance ratio (CRR) [19,20]. Lastly, the correlation of field measurements and profiling also provides information for the assessment of soil properties such as the unit weight [21] and stress history [22].

### 2.1. SASW Testing

**Figure 2:**
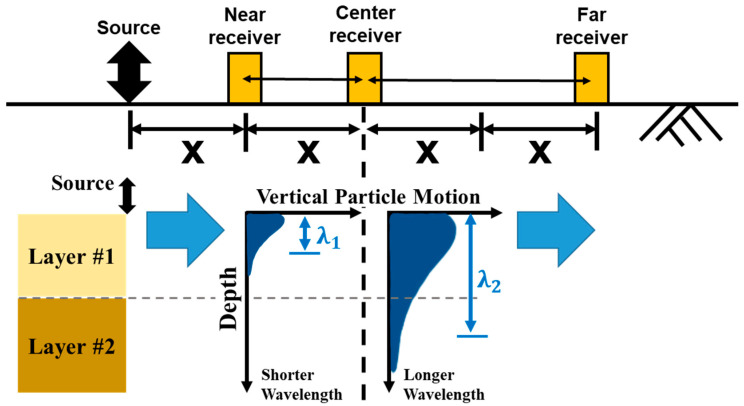
SASW testing configuration and surface waves with different wavelengths (***λ*1** and ***λ*2**) when sampling a layered system.

The SASW test was initially introduced in the 1980s [23,24]. SASW testing has been widely implemented for decades in a variety of sites due to the benefits of accuracy and the ability to test in a variety of field conditions [25,26,27] and for different purposes [28,29,30]. Figure 2 shows Rayleigh wave velocity propagation in a multi-layered half-space. The SASW method uses the vertical motion of the surface Rayleigh waves generated using a hammer or vibroseis, depending on the specific requirements of the site investigation [31]. Shorter-wavelength data are captured closer to the source, and longer-wavelength data are captured farther from the source. In other words, short receiver spacings (near receiver–center receiver) enable shallow depth profiling close to the source, and longer receiver spacings (center receiver–far receiver) enable deep depth profiling farther from the source. Each receiver spacing generates an individual dispersion. The test runs by increasing the distance between the source and the three receiver pairs while maintaining the distance ratio [32]. Typically, the spacing of the receiver equals the maximum profiling depth. Therefore, the SASW test continues with increasing spacing until the receiver spacing satisfies the depth of interest.

**Figure 3:**
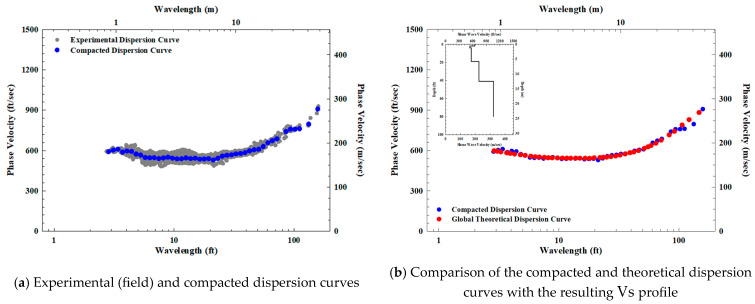
SASW data analysis procedure: (**a**) experimental (field) and compacted dispersion curves; (**b**) comparison of the compacted and theoretical dispersion curves with the resulting Vs profile.

After data collection at the site, the first phase of the SASW analysis process involves generating a field dispersion curve. This step combines the separate dispersion curves to produce an experimental dispersion curve depicting the site characteristics. The experimental dispersion curve is then averaged into a compacted dispersion curve through moving average calculation (Figure 3a). Finally, the analysis is completed by finding the theoretical dispersion curve from the Vs profile that best matches the compacted dispersion curve, as shown in Figure 3b [33,34].

### 2.2. Downhole Testing

Downhole testing measures the seismic waves in subsurface layers to assess the geological characteristics of the ground. This method helps to understand the stability and behavior of underground structures and evaluate seismic risks. The advantage of downhole testing is its precision, which allows for distinguishing layers at the test site. Moreover, compressional waves (P-waves) and shear waves (S-waves) can be measured in this test system. Furthermore, the correlation between the shear wave velocity (Vs) and the compressional wave velocity (Vp) can be utilized to determine the Poisson ratio of the subsurface. The last advantage of downhole testing lies in finding the water table depth since the water has a value of Vp = 1500 m/s.

**Figure 4:**
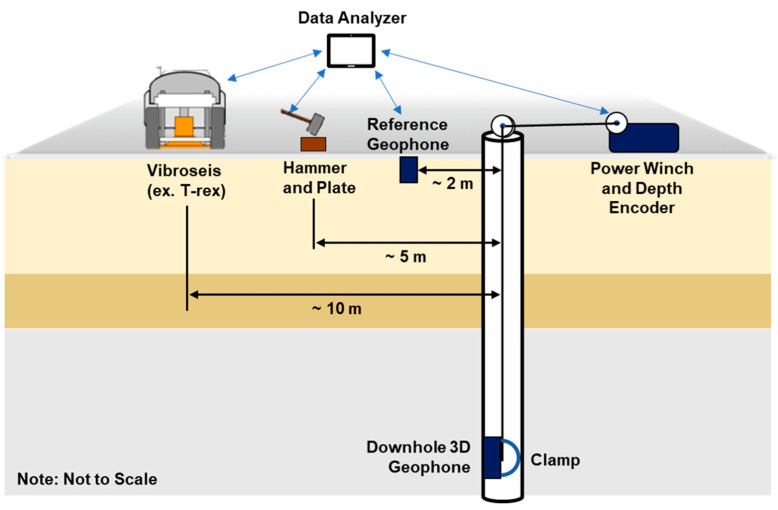
Generalized field setup for downhole seismic measurements.

In the downhole test, a 3D sensor is lowered into a borehole, as illustrated in Figure 4 [35]. The measurement interval is determined according to the purpose of the test, and the test runs based on this value. During each measurement, the sensor is pushed into the wall using an air tube or instrument clamp, and then the waves generated by the source are recorded. There are two sources: impact sources, such as a hammer, or a sweep-type source from vibroseis, such as the T-rex illustrated in Figure 4. The T-rex can vibrate in three axial directions (vertical and two horizontal) for shaking [36]. P-waves are generated by striking the plate vertically or shaking the vibroseis, while S-waves are generated by striking the plank horizontally or shaking the vibroseis. For S-wave measurements, waves in opposite directions create two inverted data. Testing in two directions is preferred for S-waves since tracking data at intersecting or opposing points with intersecting data facilitates analysis.

For data analysis purposes, the corrected travel time and depth are required. The travel time is adjusted using the following equation:

(1) t_corrected = D × t_measured/L

where

*t_corrected* = corrected travel time;

*D* = depth of receiver location;

*t_measured* = measured travel time; and

*L* = direct distance from source and receiver.

**Figure 5:**
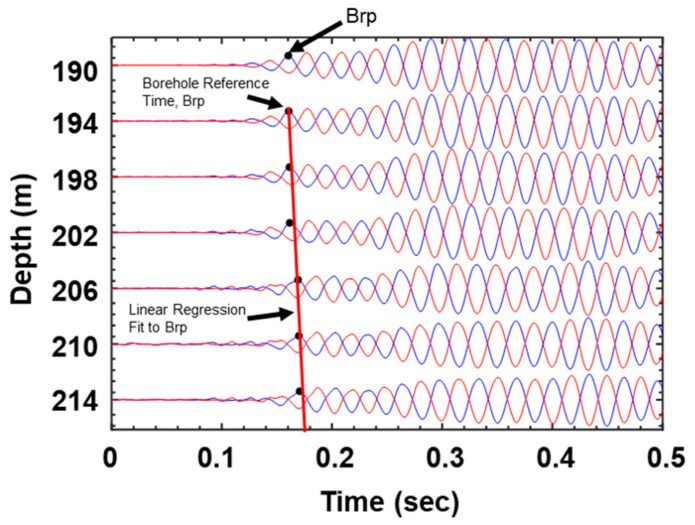
Downhole P waveforms generated with the source.

The slope of the data can help to identify the distinct layers. If the depth ranges contain the same slope, they represent a single layer, and the slope value represents the velocity of this layer. Currently, downhole analysis techniques have been improved through the SABW method [37]. Figure 5 shows an example plot of a scaled waterfall diagram with filtered P waveforms from the vertical receiver geophone. The testing depths range from 156 to 180 m. Red circles indicate travel time “picks” (Brp) on the time axis. The initial linear regression line (red line) fitted to the Brp indicates a zone with a constant-velocity layer.

### 2.3. Crosshole Testing

Crosshole testing is a technique used for underground exploration to analyze the propagation of waves in the subsurface and evaluate ground characteristics. This technique involves drilling two or more boreholes or measurement points and then emitting waves from one point and measuring them at another. Engineers analyze the collected data to understand the speed of P- and S-waves, which helps them to elucidate the ground characteristics. Adjusting the distances and depths between holes allows engineers to obtain information regarding different ground depths.

Crosshole testing offers several advantages over other geophysical methods. Firstly, it provides highly accurate and reliable results due to direct measurements between boreholes, minimizing uncertainties associated with surface measurements or indirect methods [38]. This technique also offers excellent depth resolution, allowing engineers to assess subsurface layers at various depths accurately. Measurements can also be gathered along multiple inclined ray paths, which can be processed together to render a tomographic image of the cross-section [39,40]. The problem of isotropy affecting the test results has also been addressed by considering waveform tomography [41].

However, crosshole testing requires a minimum of two boreholes, which significantly increases the cost compared to other tests. Moreover, the deeper the borehole, the less accurate the orientation, which makes it difficult to determine the exact distance between sensors. Therefore, crosshole testing generally becomes the preferred method when precise geotechnical investigations at depths of 30 m or less are required. Furthermore, the boreholes should be aligned in a straight line to facilitate precise measurements. Refraction paths in stiffer layers are a common source of error in crosshole tests [42]. Therefore, to avoid this issue, the distance between the receivers should be kept short.

**Figure 6:**
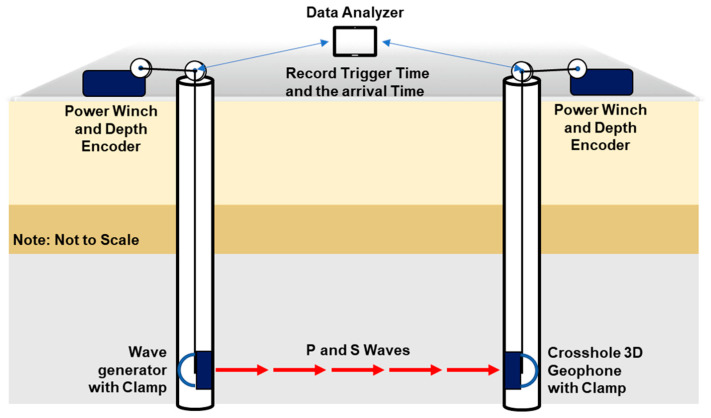
Crosshole testing setup using two boreholes.

As depicted in Figure 6, crosshole testing involves one borehole containing the source that generates the signal and one or two boreholes containing the 3D receiver [43]. These sensors transmit and receive signals during the testing process, similarly to downhole testing. Depending on the test purpose and depth, the source and 3D sensor must be lowered into the borehole. The source and sensor are then fixed in place using air tubes or instrumentation clamps, and two receivers record the signals generated by the source. The signals travel through the subsurface medium and are influenced by its properties. By determining the measured distance and the time that it takes to reach the receiver, the wave velocity can be calculated by dividing the distance by the time. The crosshole data provide information on the subsurface characteristics, such as the P- and S-wave velocities, and we can calculate the Poisson ratio from these P- and S-wave velocities.

### 2.4. Suspension Logging (PS Logging) Testing

Suspension logging, also known as PS logging, is a method that determines the shear wave velocity (Vs) and compressional wave velocity (Vp) profiles as a function of depth. This method involves measuring the time that it takes for a wave to travel upwards through the soil or rock column in a single borehole to determine the wave speed of a 1 m segment. The data obtained from this method provide information on subsurface characteristics, such as the P- and S-wave velocities, and we can calculate the Poisson ratio from these P- and S-wave velocities.

**Figure 7:**
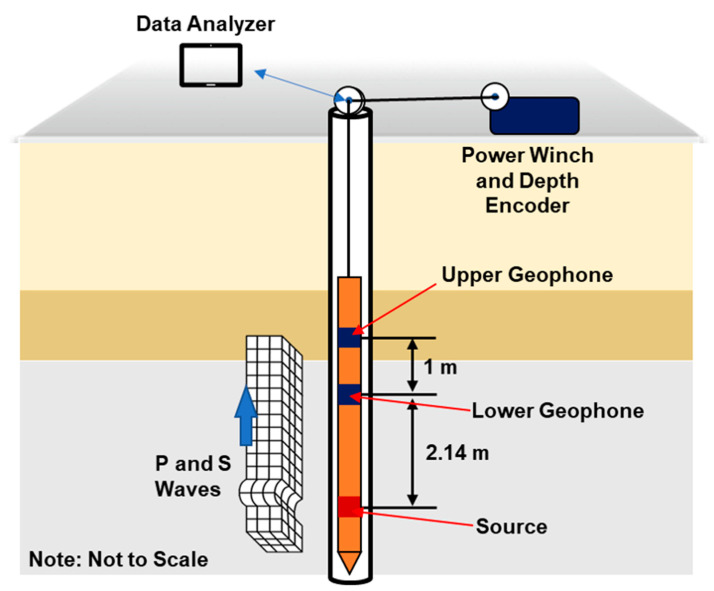
Suspension logging test setup.

A typical suspension PS logging system consists of a borehole probe with a cable and a control/recording device, as illustrated in Figure 7. The probe has a source and two 3D receivers placed at a specific distance from each other [16]. Two receivers capture the signal created by the source. The suspension system probe includes a reversible-polarity solenoid source that generates horizontal shear wave (SH) and compressional wave (P) signals. The probe moves evenly in the borehole, collecting wave data at all depths. In a study, the Vs measured by suspension logging testing showed a less conservative value than other seismic methods due to the effect of grouting between the PVC case and borehole wall [43]. Despite its drawbacks, the suspension PS logging system offers a unique opportunity in petroleum engineering for deep exploration, and the benefits include the fact that there is no restriction on the source. This distinct feature sets it apart from other methods, with potential for further deep earth exploration.

## 3. Area of Data Acquisition

**Figure 8:**
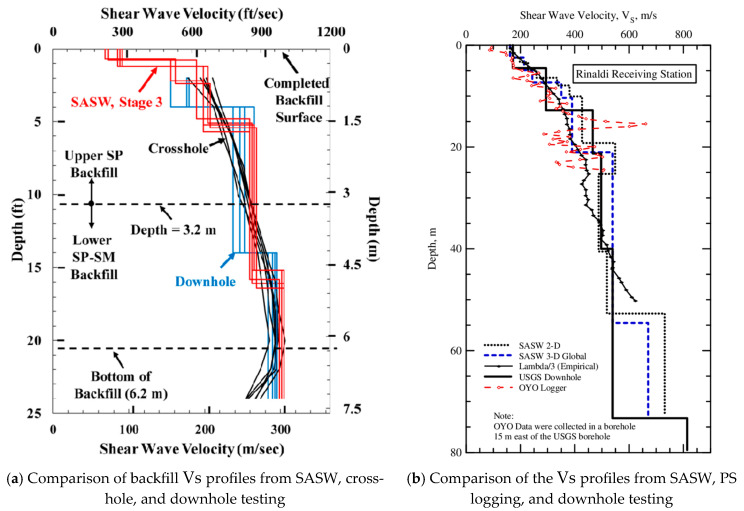
Comparison of Vs profiles from (**a**) SASW, crosshole, and downhole testing at a uniform site [38] and (**b**) SASW, PS logging, and downhole testing at a partially uniform site [39].

Generally, when comparing the results of geotechnical tests performed on uniform sites, they show very similar results, as shown in Figure 8a [44,45]. These sites often warrant accurate geotechnical investigation to understand the ground stability and allow more accurate geographic visualization. However, comparing the seismic test results from complex sites may lead to confusion due to the lack of consideration of the actual range of the test measures. For instance, the SASW test determines the Vs value by using an average value from a wide area, whereas the suspension logging test targets only one meter of target depth. Hence, comparing tests such as SASW and PS logging in a non-uniform site may be inappropriate. Figure 8b also compares the Vs profiles obtained from SASW, downhole, and suspension logging tests conducted at the same site [46]. In the comparison example, the Vs profiles present similar Vs values within a shallow depth range, but, beyond 50 m, a significant difference in the Vs values is found. Differences in the measurement range usually cause these significant differences in the Vs values.

Different types of tests play different roles. Point-based tests such as downhole and crosshole are useful for detailed geotechnical investigations of small areas, while area-based tests such as SASW are helpful for seismic design or the comprehensive evaluation of large areas. Understanding the information obtained from the various tests can be critical for accurate subsurface visualization. In particular, it is crucial to understand the area utilized for data collection, which is often disregarded. This section provides insights into the area used for data measurement in each test.

### 3.1. Area of Data Acquisition in SASW Testing

The SASW test collects data at different depths by placing an energy source and receivers on the testing array and gradually varying the distance between the receivers and the source. The SASW test utilizes data collected on an area basis. The data contain two axes: one for surface values and the other for depth values. The surface axis equals the receiver spacing value, making it easy to determine, while the depth information can be more challenging as it depends on the site characteristics.

**Figure 9:**
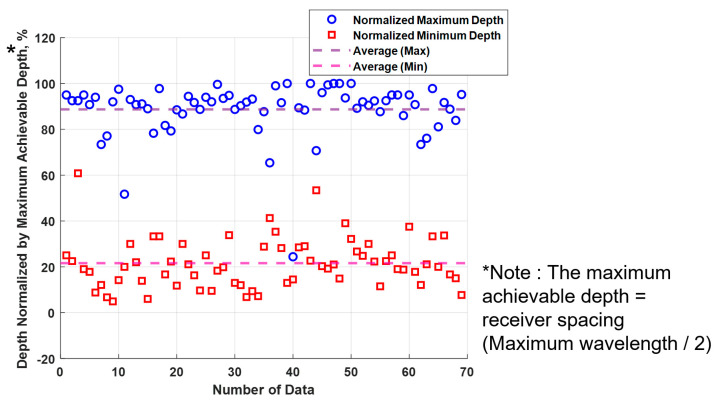
Maximum and minimum depth data for 69 SASW data normalized by the length of receiver spacing.

To address this challenge, the average range of depths used for the analysis, relative to the receiver spacing, was calculated based on existing test data and is presented in Table 1. The average and median depths, normalized by the receiver spacing, were calculated using real SASW testing data. Due to the limited dataset, a straightforward statistical model was adopted to define the normalized range. This approach was chosen to ensure ease of interpretation and applicability in practical geotechnical investigations. A normalized depth range provides a clearer basis for comparison across different datasets. The calculation utilized data from seven different SASW test arrays from three different sites. Figure 9 also shows the maximum and minimum depths of the 69 SASW data normalized by the receiver spacing, along with their average values. The values in the figure and table indicate that the depth ranges are similar across the board, except for a few outliers. As presented in the table and figure, the average utilization of the data ranges from 21.6% to 88.7% of the receiver spacing length from 69 data. Median values were also calculated to avoid the potential influence of outliers, resulting in 20.0% to 91.7% of the data being utilized, showing no significant difference from the average value. Additionally, the 95% confidence intervals for the maximum and minimum values were considered, but no significant difference was found. In conclusion, the results showed that, by performing the SASW test, approximately 20% to 90% of the receiver spacing length could be expected for the depth ranges. Outliers were not explicitly removed, as their occurrence was minimal, and the differences between the mean and median values were insignificant, indicating that outlier removal was unnecessary in this case. This was primarily due to the quality control measures applied during field testing, where the coherence function was used to pre-filter low-quality data before averaging the final dataset.

The datasets used in this study were collected from four different sites, each representing distinct geological conditions: Site A represented typical natural ground with variable subsurface conditions; Site B was a compacted fill site primarily composed of sandy silt; Site C featured a shallow soil layer overlying bedrock; and Site D was a reclaimed site composed of sandy silt.

Among these, Sites A and C represented natural, heterogeneous subsurface conditions, whereas Sites B and D were relatively homogeneous. The comparison of the results across these sites revealed that the detection sensitivity did not show significant variations between the different geological conditions. However, in highly heterogeneous subsurface environments, wave propagation constraints are expected to have a greater impact, potentially affecting the reliability of the results.

**Figure 10:**
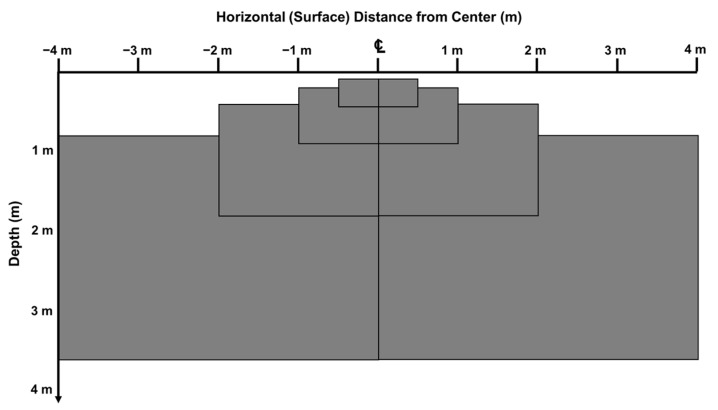
The areas of data collected using the improved SASW method (3.6 m depth profiling).

Based on the previous calculations, a data acquisition area for geotechnical information down to a depth of approximately 3.5 m was designed utilizing the improved SASW method, and this is illustrated in Figure 10 [32]. In Figure 2, the test setup illustrates a one-directional SASW measurement. The test provides depth data proportional to the distance from the center receiver, with the left side yielding data at x and the right side at 2×. As a result, the data collection becomes asymmetrical with respect to the center. Unlike conventional SASW tests, the improved SASW method applies a two-way testing approach. This enhances the accuracy by effectively doubling the dataset while requiring only approximately 30% additional testing time. Additionally, the two-way measurement allows for the identification of lateral differences, improving the reliability of the subsurface characterization. The simulation was conducted with receiver spacings of 0.5 m, 1 m, 2 m, and 4 m, resulting in a calculated test area of 24.5 m^2^. This area provided information at depths ranging from approximately 0.17 m to 3.6 m. As shown in the figure, the utilized area forms a symmetrical triangular shape, characteristic of the improved SASW method.

### 3.2. Area of Data Collection in Downhole Testing

**Figure 11:**
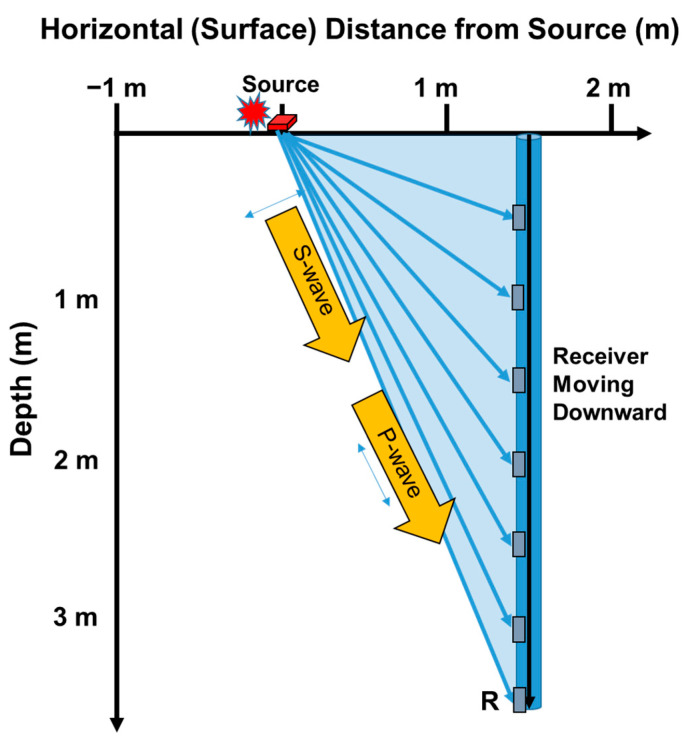
The areas of data collected using downhole testing (3.6 m depth profiling).

For downhole tests, data analysis requires depth adjustments based on the angle of the ray path to ensure accurate results. The longest ray path used for actual measurement extends from the source to the receiver (R), as shown in Figure 11. Consequently, the total data acquisition area corresponds to the surface area of the blue triangle formed by this longest ray path (Figure 11). The distance between the source and the borehole typically varies depending on the site conditions and the type of source used. In the example illustrated, a distance of 1.5 m between the source and the borehole is utilized for shallow depth measurements. For a measurement targeting a depth of approximately 3.6 m, the utilized area is calculated to be 2.7 m^2^. While increasing the distance between the source and the borehole can expand the data acquisition area, it may also alter the ray path direction, potentially compromising the accuracy of the test. For this reason, expanding the distance beyond the necessary limits is not recommended.

### 3.3. Area of Data Collection in Crosshole Testing

The crosshole test utilizes a straight ray path, making the areas covered by the test relatively straightforward to define. Crosshole tests are conducted with depth intervals similar to those in downhole tests; however, the intervals are typically small enough to be considered negligible for this study. The total data acquisition area is calculated as the rectangular area defined by the distance between the boreholes and the depth of the test.

**Figure 12:**
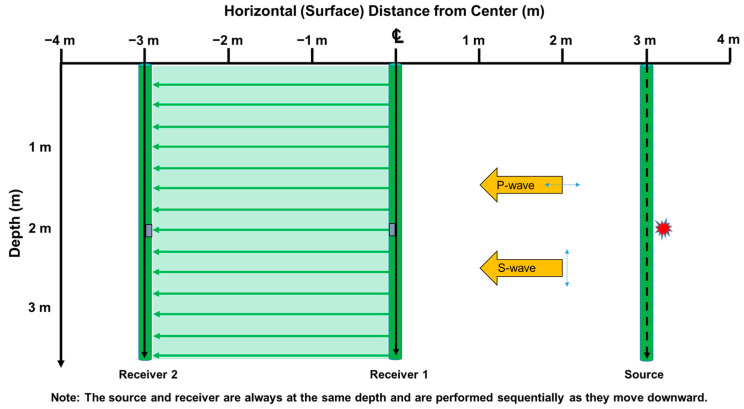
The areas of data collected using crosshole testing with 3 boreholes (3.6 m depth pro-filing).

In standard practice, crosshole tests primarily use data collected between Receiver 1 and Receiver 2. However, if the signal trigger time is recorded, the data between the source and Receiver 1 can also be included in the analysis for more comprehensive results. Figure 12 depicts the area used for data acquisition during a crosshole test performed to a depth of 3.6 m, with boreholes spaced 3 m apart. In this example, the total measurement area is calculated to be 21.6 m^2^.

### 3.4. Area of Data Collection in PS Loggging Testing

**Figure 13:**
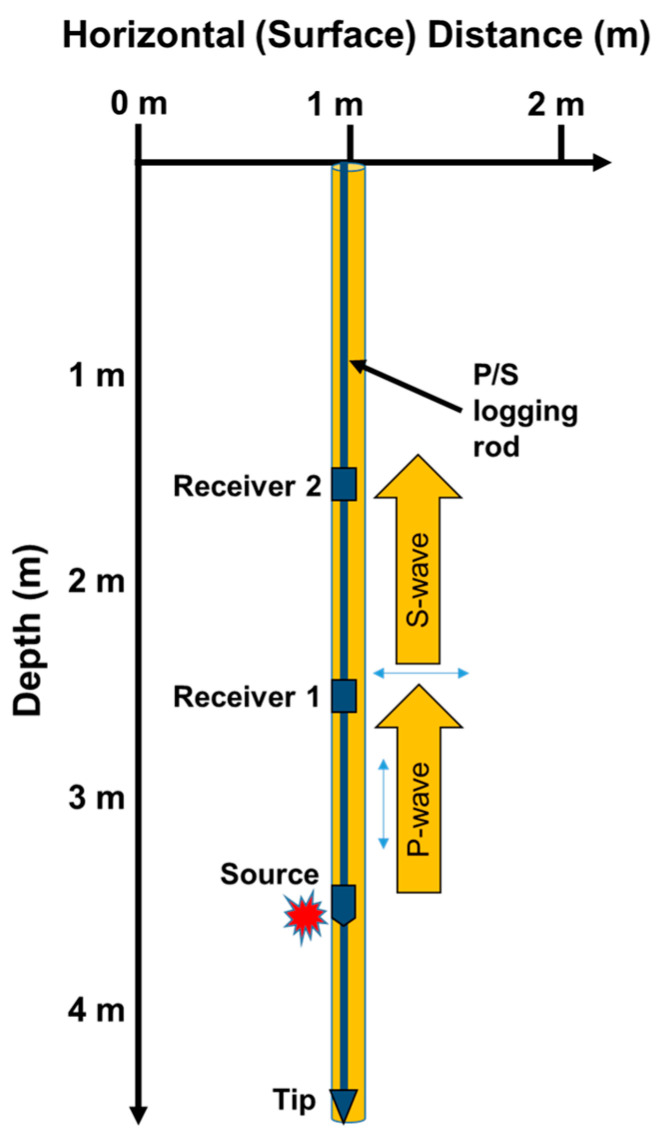
The areas of data collected using PS logging testing (3.6 m depth profiling).

The area covered by PS logging data collection is relatively straightforward to define. In PS logging, the source and receivers are mounted on the same rod, resulting in a ray path that runs along the adjacent wall of the borehole. As a result, the cross-sectional area of the borehole is approximately equal to the data acquisition area. Unlike crosshole and downhole tests, PS logging requires deeper penetration to achieve the desired profile. For instance, to obtain a 3.6 m profile, the rod must penetrate approximately 4.6 m, as the tip of the rod sits about 1 m below the source (Figure 13). Assuming a borehole diameter of 10 cm, the data acquisition area for this test is calculated to be 0.36 m^2^, allowing for a direct comparison with the results of other tests. Furthermore, it can be assumed that this test covers a range that is comparable to that of non-seismic tests such as the SPT or CPT, given its localized data acquisition scope.

### 3.5. Comparisons of the Area of Data Collection Using All Four Tests

**Figure 14:**
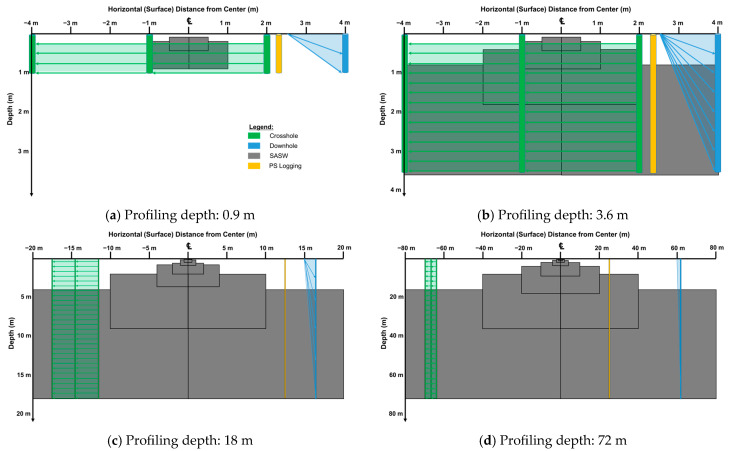
Comparisons of the area of data collection using four different tests (SASW, crosshole, downhole, and PS logging) when profiling at (**a**) 0.9 m, (**b**) 3.6 m, (**c**) 18 m, and (**d**) 72 m.

This sub-section provides a comparative analysis of the data acquisition areas used in four seismic tests: SASW, crosshole, downhole, and PS Logging. The data acquisition areas were calculated and compared at profiling depths of 0.9 m, 3.6 m, 18 m, and 72 m, as shown in Figure 14. Figure 14a–d illustrate the areas utilized by each test at these four depths: 0.9 m (Figure 14a), 3.6 m (Figure 14b), 18 m (Figure 14c), and 72 m (Figure 14d).

The SASW test used four different receiver spacing sets for the calculation: 0.5 and 1 m for sets 1; 2 and 4 m for set 2; 10 and 20 m for set 3; and 40 and 80 m for set 4. Only receiver spacing set 1 was utilized for 0.9 m profiling; set 1 and set 2 were utilized for 3 m profiling; and sets 1 to 3 were utilized for 18 m profiling. Finally, all four sets were utilized for 72 m profiling. The crosshole test was considered in two cases: one with three boreholes and 3 m spacing and the other with three boreholes and 5 m spacing. The downhole test considered two distances: 1.5 m and 5 m from the source to the borehole.

The results in Figure 14a indicate that the crosshole test utilizes the largest area for data collection at shallow depths. However, as the profiling depth increases, the SASW test covers a more extensive area due to its use of averaging over a broader range. In particular, Figure 14d highlights the challenges of comparing SASW data to those of other tests in non-uniform sites. The non-linear behavior and larger data acquisition area of SASW at greater depths can complicate direct comparisons with other tests, emphasizing the need for careful interpretation when dealing with non-uniform subsurface conditions. This analysis is based on simulated comparisons and aims to provide insights into the relative performance and coverage of the four seismic tests under varying conditions.

For more specific comparisons, the calculations for the area utilized by depth in each test were summarized (Table 2). As summarized in the table, when conducting a geotechnical survey to a depth of 72 m, the differences in the area of data collection are significant. For instance, for 3-m-spaced crosshole tests, the difference is 23 times that of SASW, while, for 5-m-spaced crosshole tests, it is 14 times. Similarly, the difference in downhole tests is significant, being approximately 180 times greater than in SASW for a source-to-borehole distance of 1.5 m and about 55 times greater for a source-to-borehole distance of 5 m. Lastly, in the case of PS logging, which uses a similar area as in the CPT and SPT, the difference is approximately 1360 times compared to the SASW test. During the modeling of data obtained from SASW tests, the receiver spacings were increased by approximately four times the original distance to determine the test area. This increase in spacing ensured that there was sufficient overlap in the test area. The spacing between sets can be adjusted based on the specific test purpose and time constraints.

The results highlight that comparing geophysical test results from complex sites can be challenging due to the need to account for the actual measurement range. Each type of test serves a specific purpose, making it essential to understand their applications and limitations. Point-based tests, such as downhole and crosshole, are well suited for detailed geotechnical investigations in small, localized areas, while area-based tests like SASW are more effective for seismic design and broad-scale site assessments. The choice between these methods depends on the engineering objectives, site conditions, and budget constraints. For example, in heterogeneous subsurface conditions, as often encountered in complex ground environments, a single method may not provide sufficient accuracy. In such cases, integrating multiple geophysical tests can enhance the reliability of subsurface characterization.

A hybrid approach, using SASW for large-area site mapping and downhole or crosshole testing for localized high-resolution validation, maximizes both the efficiency and accuracy. Additionally, foundation investigations for high-rise buildings favor borehole-based tests for detailed profiling in a limited area, while seismic design applications rely on SASW to capture long-wavelength wave propagation. In waste landfill investigations, where borehole drilling poses risks due to toxic gas leakage, SASW and other non-intrusive methods offer a safer alternative. These examples illustrate that no single method dominates—the optimal approach varies based on the engineering requirements, site characteristics, and practical constraints.

## 4. A Case of Defect Detection Based on an Understanding of the Test Measurement Area

This test aimed to non-destructively assess whether a reduction in strength occurred within approximately 10 m of the dam spillway slope. As the study site was a dam structure, only non-destructive testing methods were applicable to avoid compromising the structural integrity. The SASW test served as a preliminary investigation tool, allowing for the identification of potential weak zones that required further detailed assessments. This approach enabled targeted follow-up investigations, minimizing unnecessary interventions while ensuring structural safety. For this dam, the upper 1.8 m consisted of a concrete layer, while the lower layers comprised high-strength bedrock. The test utilized a hammer and a vibration control truck, commonly referred to as a seismic energy source truck. This truck, known for its superior mobility compared to other large seismic sources, is particularly useful for field tests in areas with limited accessibility [31]. The hammer was employed to acquire high-frequency signals for short-wavelength data, whereas the seismic energy source truck was used to generate low-frequency signals for long-wavelength data collection.

**Figure 15:**
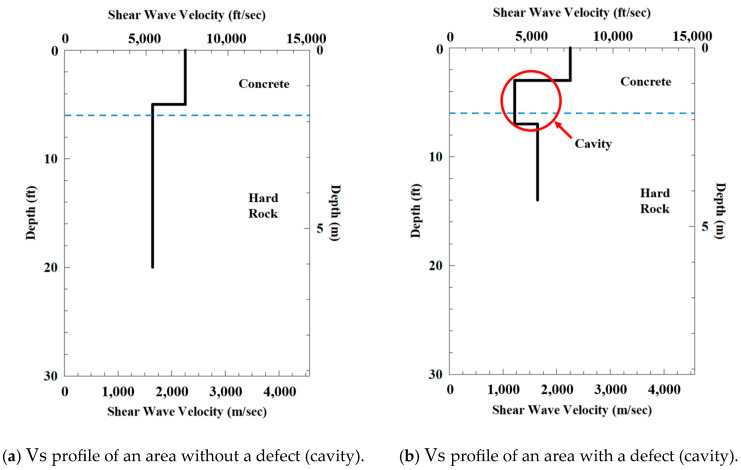
An example of defect detection using the SASW test.

Figure 15 illustrates the results of the improved SASW test. A notable difference appeared between the data collected from the left area (Figure 15a) and those from the right area (Figure 15b) relative to the test center. Figure 15a displays the Vs profile of an area without defects, confirming that the upper 1.6 m consisted of a concrete layer, with dense bedrock underlying this layer. In contrast, Figure 15b shows the Vs profile of an area with a defect. The results revealed an intact concrete layer up to 1 m, followed by a zone of reduced strength that was approximately 1 m thick between 1 and 2 m. Below this layer, the profile once again demonstrated the strength of the bedrock.

These findings prompted a detailed investigation of the right area relative to the test center. The location of the defect was deduced based on the area covered by the SASW method. Since the defect was detected to the right of the test center and at a depth between 1 and 2 m, it was constrained to the range of 0 to 4 m, as indicated in Figure 14b. An initial investigation was conducted at the midpoint, 2 m, leading to the successful identification of the defect at an approximately 1 m depth at this location.

The spatial resolution of SASW is primarily governed by the dispersion characteristics of surface waves, where longer wavelengths, corresponding to deeper layers, tend to average out small-scale anomalies. Consequently, while SASW is effective in capturing broad-scale variations in subsurface stiffness, it may fail to resolve minor voids, thin weak zones, or localized fractures. However, SASW remains capable of detecting voids and weak zones that are significant enough to impact structural integrity and safety. In the specific case analyzed in this study, a defect with a 1 m size was successfully identified within a 5 m investigation depth. Although further studies are necessary to evaluate the detection sensitivity under varying conditions, these findings indicate that SASW was able to detect a weak zone at approximately 20% of the profiling depth in this case. This demonstrates that, despite its inherent resolution limitations, SASW can be effective in identifying critical subsurface anomalies that may influence geotechnical design and safety assessments.

This case highlights the importance of understanding the measurement area covered by the test, which significantly enhances the probability of accurate detection. If a point-based method, such as the CPT, had been employed instead of the area-based SASW method, the detection process would likely have faced substantial challenges. The results demonstrate that the SASW method was not only well suited for this site and its objectives but also that a clear understanding of the measurement scope significantly improved the efficiency and effectiveness of the investigation. It is worth noting that the SASW test generates averaged data, which prevents the strength in regions with cavities from dropping to zero. Nevertheless, the results clearly demonstrate the capabilities of the improved SASW method to effectively detect and identify zones with reduced strength, highlighting its potential as a non-destructive diagnostic tool for structural assessments.

## 5. Conclusions and Discussion

Geotechnical investigations involve various methods and play an essential role in safely designing and constructing structures. Understanding the test results and proper test application will support the creation of accurate subsurface information. In addition, comparison without considering the data acquisition area often leads to interpretation errors. Therefore, this study aimed to determine the area utilized for geotechnical seismic testing based on the depth of data acquisition to enhance the application and data.

This study focused on the area used for data acquisition in seismic wave tests such as SASW (a non-destructive surface wave test) and borehole tests like crosshole, downhole, and PS logging. In addition, the recent trends of each test were reviewed, and the most recent techniques were utilized for the study. The findings of this study are summarized below.

(1) Geotechnical tests conducted on uniform sites usually yield similar results. However, comparing the seismic test results from complex sites may lead to confusion because the actual range of the test measures is not considered. Different types of tests have different roles, and it is essential to understand and apply them appropriately. Point-based tests, such as downhole and crosshole, are practical for detailed geotechnical investigations of small areas. On the other hand, area-based tests, such as SASW, are helpful in searching defects or in the comprehensive evaluation of large areas.
(2) Since the SASW test utilizes surface waves, determining the area is more challenging than in other tests. The SASW test utilizes data collected on an area basis. The surface axis equals the receiver spacing value, making it easy to determine, while the depth information can be more challenging as it depends on the site characteristics. Therefore, we calculated the depth utilized by the test on average using 69 SASW data. As a result, the mean and median were not significantly different. Therefore, the average (20 to 90% of the receiver spacing) was used to estimate the depth.
(3) To determine the measurement area for different types of borehole tests, the following calculations were used. For crosshole tests, the square section formed by the borehole and the test depth was considered as the measurement area. For downhole tests, the right triangle formed by the source and the receiver position at the final depth was considered as the measurement area. For PS logging, the wall surface of the borehole was used as the measurement area, and its approximate area was calculated accordingly.
(4) The area used for data collection in different depth scenarios (0.9, 3.6, 18, and 72 m) was calculated for each test. The results showed that, for shallower depths (0.9 m), crosshole testing covered the largest area for geotechnical investigation. However, as the depth increased, the SASW test covered a significantly larger area than the other tests. In the extreme case of 72 m profiling, the SASW test covered an area approximately 1360 times larger than that of PS logging.
(5) Through a case study, the improved SASW method was demonstrated as a non-destructive approach to detecting defects on a dam spillway. The test revealed differences in the Vs profiles, effectively identifying zones with reduced strength and cavities. A clear understanding of the measurement area improved the detection efficiency, highlighting the method’s suitability for subsurface investigations.

A detailed statistical approach may optimize the use of different geotechnical tests, significantly improving their accuracy for future studies.

## Figures and Tables

**Table 1:** The average and median depths normalized by the receiver spacing calculated from SASW testing data.

Site	Array Name	Number of Test Sets	Normalized Minimum Depth (Average), %	Normalized Maximum Depth (Average), %	Normalized Minimum Depth (Median), %	Normalized Maximum Depth (Median), %
A	A-1	9	19.7%	89.1%	17.8%	92.5%
	A-2	6	17.7%	85.5%	17.1%	90.9%
B	B-1	5	23.5%	85.1%	22.3%	81.7%
	B-2	6	18.6%	91.2%	18.7%	91.8%
C	C-1	14	20.1%	85.7%	16.4%	91.7%
	C-2	4	33.4%	87.1%	28.7%	88.9%
	C-3	6	24.4%	98.2%	20.7%	99.7%
D	D-1	5	23.0%	90.3%	24.8%	90.5%
	D-2	7	21.8%	89.7%	19.0%	92.5%
	D-3	7	21.1%	87.8%	20.0%	88.8%
Total	-	69	**21.6%**	**88.7%**	**20.0%**	**91.7%**
Confidence interval 95%			19.0~24.1%	85.9~91.5%	-	-

**Table 2:** Comparison of the area of data collection using all four tests (SASW, crosshole, downhole, and PS logging).

Profiling Depth, m	Crosshole (Borehole Spacing: 3 m), m^2^	Crosshole (Borehole Spacing: 5 m), m^2^	Downhole (Source to Borehole: 1.5 m), m^2^	Downhole (Source to Borehole: 5 m), m^2^	PS Logging, m^2^	SASW, m^2^
0.9 m	5.4	9	0.675	2.25	0.09	1.5
3.6 m	21.6	36	2.7	9	0.36	24.5
18 m	108	180	13.5	45	1.8	611.7
72 m	432	720	54	180	7.2	9811.7

## Data Availability

The original contributions presented in this study are included in the article, and further inquiries can be directed to the corresponding author.
